# Genomic profiling of a Hepatocyte growth factor-dependent signature for MET-targeted therapy in glioblastoma

**DOI:** 10.1186/s12967-015-0667-x

**Published:** 2015-09-17

**Authors:** Jennifer Johnson, Maria Libera Ascierto, Sandeep Mittal, David Newsome, Liang Kang, Michael Briggs, Kirk Tanner, Francesco M. Marincola, Michael E. Berens, George F. Vande Woude, Qian Xie

**Affiliations:** Molecular Oncogenesis and Targeted Therapy, Laboratory of Molecular Oncology, Van Andel Research Institute, 333 Bostwick AVE NE, Grand Rapids, MI 49503 USA; Laboratory of Molecular Oncology, Van Andel Research Institute, Grand Rapids, MI USA; Department of Transfusion Medicine, Infectious Disease and Immunogenetics Section, Clinical Center, Trans-National Institutes of Health Center for Human Immunology, National Institutes of Health, Bethesda, MD USA; Department of Oncology, Johns Hopkins University, Baltimore, MD USA; Departments of Neurosurgery and Oncology, Karmanos Cancer Institute, Wayne State University, Detroit, MI USA; Vertex Pharmaceutical Inc., Boston, MA USA; Woodland Pharmaceuticals, Shrewsbury, MA USA; Research Branch, Sidra Medical and Research Center, Doha, Qatar; Translational Genomics Research Institute, Phoenix, AZ USA

**Keywords:** Predictive signature, Hepatocyte growth Factor, MET, Glioblastoma, Targeted therapy

## Abstract

**Background:**

Constitutive MET signaling promotes invasiveness in most primary and recurrent GBM. However, deployment of available MET-targeting agents is confounded by lack of effective biomarkers for selecting suitable patients for treatment. Because endogenous HGF overexpression often causes autocrine MET activation, and also indicates sensitivity to MET inhibitors, we investigated whether it drives the expression of distinct genes which could serve as a signature indicating vulnerability to MET-targeted therapy in GBM.

**Methods:**

Interrogation of genomic data from TCGA GBM (Student’s *t* test, GBM patients with high and low HGF expression, p ≤ 0.00001) referenced against patient-derived xenograft (PDX) models (Student’s *t* test, sensitive vs. insensitive models, p ≤ 0.005) was used to identify the HGF-dependent signature. Genomic analysis of GBM xenograft models using both human and mouse gene expression microarrays (Student’s *t* test, treated vs. vehicle tumors, p ≤ 0.01) were performed to elucidate the tumor and microenvironment cross talk. A PDX model with EGFR^amp^ was tested for MET activation as a mechanism of erlotinib resistance.

**Results:**

We identified a group of 20 genes highly associated with HGF overexpression in GBM and were up- or down-regulated only in tumors sensitive to MET inhibitor. The MET inhibitors regulate tumor (human) and host (mouse) cells within the tumor via distinct molecular processes, but overall impede tumor growth by inhibiting cell cycle progression. EGFR^*amp*^ tumors undergo erlotinib resistance responded to a combination of MET and EGFR inhibitors.

**Conclusions:**

Combining TCGA primary tumor datasets (human) and xenograft tumor model datasets (human tumor grown in mice) using therapeutic efficacy as an endpoint may serve as a useful approach to discover and develop molecular signatures as therapeutic biomarkers for targeted therapy. The HGF dependent signature may serve as a candidate predictive signature for patient enrollment in clinical trials using MET inhibitors. Human and mouse microarrays maybe used to dissect the tumor-host interactions. Targeting MET in EGFR^*amp*^ GBM may delay the acquired resistance developed during treatment with erlotinib.

**Electronic supplementary material:**

The online version of this article (doi:10.1186/s12967-015-0667-x) contains supplementary material, which is available to authorized users.

## Background

Glioblastoma (GBM) exhibits infiltrative tumor growth, a feature which is a prominent cause of mortality [1, 2]. Despite progress in understanding the molecular mechanisms of GBM invasiveness, there remains a lack of effective therapeutic approaches. MET activation leads to RTK/RAS/PI3 K pathway signaling [3–5] and is associated with a GBM mesenchymal phenotype, which is more invasive and associated with shorter patient survival [5, 6]. These traits of GBM argue for the use of drugs directed against MET for treating certain GBM patients.

The epidermal growth factor receptor (EGFR) is frequently amplified, overexpressed, and/or variantly spliced (EGFR*vIII*) in GBM [4], therefore is being evaluated extensively as a promising target for treating GBM. However, the effects of EGFR-targeted therapy remains inclusive [7]. Although at preclinical level EGFR inhibitor alone or in combination with radiation therapy both showed efficacy in treating GBM tumors, clinically, no overall benefit has been observed in GBM patients treated with EGFR inhibitors [8, 9]. The major challenge of EGFR- targeted therapy is the inherent and acquired resistance, including the acquisition of secondary EGFR point mutations, co-activation of other receptor tyrosine kinases, such as IGFR1, MET, PDGFα/β, and uPAR [10]. Intriguingly, EGFR*vIII* is cross-activated by MET in GBM models [11] and MET inhibitors synergize with EGFR inhibitors against GBM xenografts harboring both EGFR*vIII* mutation and PTEN deletion [12]. Other concerns also include the low efficiency of EGFR inhibitor in penetrating blood brain barrier [7].

The Cancer Genome Atlas Network (TCGA) enables discovery of signatures for the molecular classification of GBM [6] as well as discerning distinct, aberrantly activated signaling pathways [4]. Recent work by Brennan et al. demonstrated that systematic genomic analyses with detailed clinical annotation, including treatment and survival outcomes, can be used to discover genomic-based predictive and therapeutic biomarkers [13]. Strategies to establish genomic signatures which predict therapeutic response at a preclinical level, if validated in follow-up patient studies, offer to improve patient selection for clinical trials and accelerate the development of targeted therapy and help realize the promise of personalized medicine.

Previously, we demonstrated that Hepatocyte growth factor (HGF)-autocrine activation is a strong molecular feature that predicts sensitivity to MET inhibitors in GBM [14]. Because GBM is a heterogeneous disease in which drug response can be influenced by different mechanisms, the expression of a single gene (i.e., HGF expression) was not expected to fully account for sensitivity to the drug; recent results from clinical trials have shown that total MET expression levels do not indicate responsiveness to MET inhibitors [15]. In this study, we attempted to extend our findings to a molecular signature that can be used as a biomarker to indicate sensitivity to MET inhibitors. Further, using both human and mouse gene expression microarrays, we studied how the microenvironment may respond to MET inhibition. Finally, we show that in GBM with EGFR amplification (EGFR^*amp*^), long-term exposure to erlotinib induces adaptive tumor growth that involves MET pathway activation, supporting the use of a combination of both inhibitors to more effectively control GBM progression.

## Methods

### Cell culture and compounds

DBM2, U251M2, U87M2 are subclones of DBTRG-MG, U251MG, and U87MG cells as described previously [16]. U118 and SF295 were from NCI-60 [14]. U87M2 and DBM2 cells were transfected with pCLPCX-MCS1 plasmid containing AP-1 transcriptional factor and firefly luciferase (Vertex Pharmaceuticals). The KCI-10-40X1 xenograft tumor line was generated from the primary tumor of a GBM patient upon surgical removal at Karmanos Cancer Institute. G116 and G91 are patient- derived xenograft (PDX) models provided by the Mayo Clinic. All studies involving human subjects and human tissues were approved by the IRB of Van Andel Research Institute. V-4084 is a MET inhibitor provided by Vertex Pharmaceutics and erlotinib was purchased through L C Laboratories (Woburn, MA).

### Kinase inhibitory assay

The inhibitory activity of V-4084 against 15 kinases was determined using the residual kinase activity of MET using a radiometric assay as described in Additional file 1: Supplementary Methods.

### 3D cell invasion assay

U87MG cells were first grown in 1 % soft agar (Sigma) for 7 days to form spheroids. Each spheroid was then selected and placed onto Matrigel in a well to attach overnight (day 0), followed by treatment with DMSO or serially diluted compounds. Images were taken after an additional 3 days under a light microscope. Triplicates were tested for each concentration.

### HGF induced proliferation assay and urokinase activity assay and Western Blot

These procedures have been published previously [14] and are detailed in Additional file 1: Supplementary Methods.

#### In vivo V-4084 and erlotinib therapeutic efficacy study

All animal studies were approved by the IACUC of Van Andel Research Institute. Subcutaneous and orthotopic [14, 16] tumor initiation were performed as previously described. The orthotopic tumor growth was measured by bioluminescence signal intensity (BLI) using a small animal optical imager AMI 1000 (Spectral Instruments Imaging, LLC). Dosing with V-4084 and/or erlotinib was delivered once daily by oral gavage for 3 weeks. Vehicles used were 0.5 % MC 400 with 0.05 % Tween 80 (for V-4084) and with 0.5 % (w/v) methyl cellulose (for erlotinib). To determine the effectiveness of treatment, the average tumor size of each group from the last measurement was analyzed with Student’s *t* test (p < 0.05).

### Genomic analysis

From either control or treated animals, tumors were harvested for gene expression profiling after 7 days of treatment with V-4084. Total mRNA were extracted using miRNeasy minikit (Qiagen, Valencia, CA). Global gene expression profiling (GSE64667) was analyzed using BRBArrayTools (http://linus.nci.nih.gov/BRB-ArrayTools.html). To identify the genes that are differentially expressed in GBM patients with high or low HGF expression, the same TCGA data sets (n = 202) was analyzed using Student’s *t*-test (p ≤ 0.00001) under the same criteria as we reported before, considering the top 10 % of GBM specimens with the highest HGF expression as tumors with HGF-autocrine activation [14]. Genes that are differentially expressed in sensitive and insensitive xenograft tumor models were analyzed using Student’s t-test (p ≤ 0.005). A combined use of human and mouse microarrays was performed to identify the genes that are differentially expressed in treated tumors (Student’s *t*-test, treated vs. vehicle tumors, p ≤ 0.01). All pathway analysis was performed using the Ingenuity Pathway Analysis system (IPA, Qiagen). To predict the sensitivity to MET inhibitor in PDX models, previously generated Agilent gene expression data from 40 patient-derived tumor xenograft (PDX) samples were obtained from the Gene Expression Omnibus (GSE39242). All further data processing and analysis was performed using the Bioconductor libraries for the R statistical framework [17]. Expression values for the 21 genes associated with the tumor sensitivity were isolated from each PDX sample. The resulting expression value matrix was organized by hierarchical clustering using the heatmap2 function with default settings.

### Fluorescence in situ hybridization (FISH)

This procedure was performed previously [14] and is detailed in Additional file 1: Supplementary Methods.

### qPCR

Quantitative real-time PCR was assayed by TaqMan Gene Expression Assays (Applied Biosystems, Foster City, CA). The -fold difference between insensitive and sensitive tumours was calculated using the comparative 2^−ΔΔCt^ [18].

### Immunofluorescence staining

KCI-10-40X1 cells were grown in 6-cm dishes with glass bottom and fixed in 4 % paraformaldehyde for 15 min. Cells were stained with antibodies as described in Additional file 1: Supplemental Methods. Imagines were taken under Zeiss model 510 confocal microscope.

## Results

### Selective MET kinase inhibition prevents HGF-autocrine-mediated GBM invasion

**Fig. 1:**
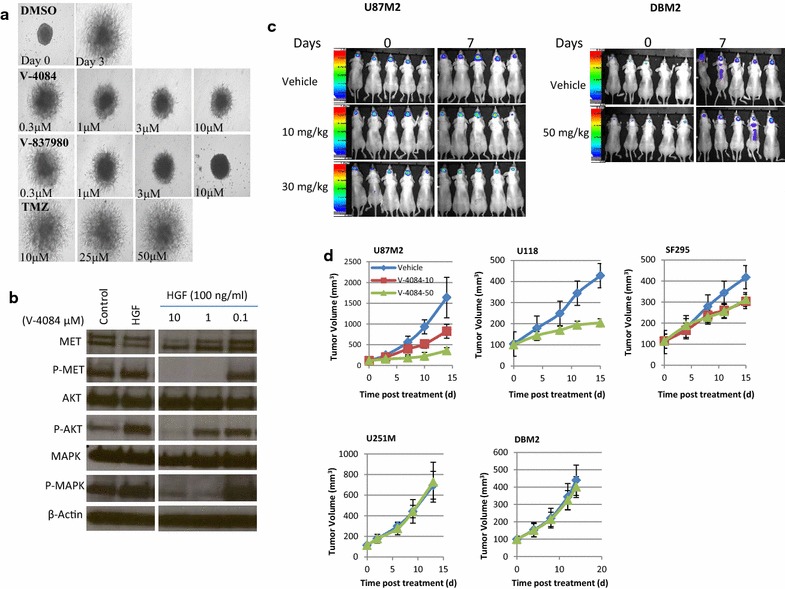
V-4084 inhibits HGF-autocrine GBM proliferation and invasion. **a** By day 3, U87M2 cells had dispersed significantly (*top panel*). While V-4084 and its derivative V-837980 significantly inhibited U87MG dispersal at 1 μM, TMZ at 50 μM failed to show any activity. **b** U87M2 cells constitutively show P-MET, P-MAPK, and activate AKT following HGF. V-4084 inhibited HGF-dependent downstream pathways (MET and MAPK) in U87MG dose-dependently. **c** To evaluate V4084 efficacy orthotopically, U87M2 cells expressing a luciferase reporter gene (U87M2Luc+) were inoculated into nude mice orthotopically, and tumor growth was monitored by BLI twice a week. V4084 at 30 mg/kg significantly inhibited tumor growth orthotopically (1 week of dosing, one dose per day; p < 0.05). DBM2 showed no response to V-4084. **d** V-4084 dose-dependently inhibited HGF-autocrine (U87M2, U118, and SF295) subcutaneous tumor growth, but had no effect against tumors without HGF expression (U251M2 and DBM2)

We previously reported that selective MET inhibitors may specifically inhibit HGF-autocrine GBM tumor growth [14]. Here, we used V-4084, a small molecule compound that selectively inhibits MET kinase activity (K_i_ = 0.025 µM; Additional file 1: Table S1), to further test inhibition of HGF-autocrine-dependent GBM invasion using U87MG malignant glioma cells. Temozolomide (TMZ), the standard first-line cytotoxic chemotherapy for GBM patients, was used as a reference treatment (Fig. 1a). V-4084 significantly inhibited U87MG cell dispersal at 3 μM. At the molecular level, V-4084 inhibited MAPK signaling at 1 μM or higher concentration, and AKT pathway between 1 and 10 μM, suggesting V-4084 targets invasion-related signaling pathways more strongly than proliferation or survival pathways. Another MET inhibitor V-837980 showed similar results, completely blocking cell dispersal at 3–10 μM. As anticipated, TMZ at 50 μM failed to show any anti-migratory activity. The efficacy of V-4084 in inhibiting tumor growth was tested against orthotopic tumors, in which a firefly luciferase reporter gene was transferred into GBM cells with (U87M2) and without (DBM2) endogenous HGF expression. V-4084 significantly inhibited U87M2 tumor growth over 7 days, while DBM2 tumor growth was unaffected (Fig. 1c). Consistent with our previous results, the HGF-autocrine tumor models U87M2 and U118 were sensitive to V-4084 in a dose-dependent manner, while DBM2 and U251M2 showed no response (Fig. 1d); SF295 cells showed modest sensitivity to V-4084. Thus, the U87M2, U118, and SF295 malignant glioma cells were determined to be models sensitive to MET inhibition, while DBM2 and U251M2 cells were used as insensitive models for further analysis. V-4084 also dose-dependently inhibited HGF induced proliferation, urokinase activity, and downstream pathway activation (see Additional file 1: Fig. S1).

### HGF-Autocrine GBMs have common genomic profiles

**Fig. 2:**
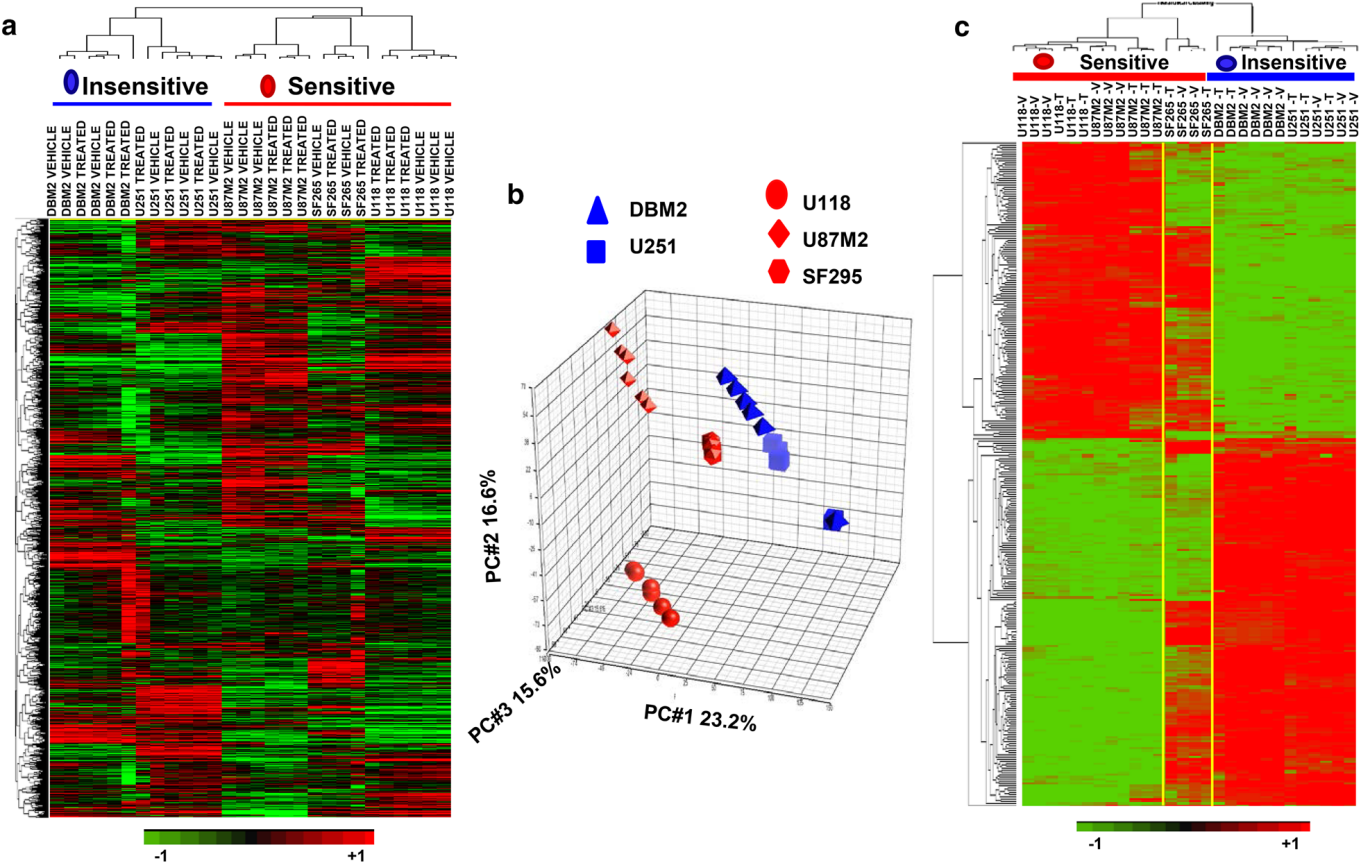
GBM models sensitive to V-4084 share common genetic profiles. **a** Unsupervised hierarchical clustering was performed on tumor samples from Fig. 1d; three tumors from each group were used for analysis. Sensitive tumors (U87M2, U118, and SF295SQ1) clustered together, away from the insensitive ones (U251M2 and DBM2). Within the most sensitive tumors (U87M2 and U118), there was a clear separation between V4084-treated and vehicle-treated samples. **b** Principal component analysis (PCA) corroborated the results shown in *panel A*. All sensitive tumors were closer to each other and farther from the insensitive tumors. Note that SF295 showed partial sensitivity to V4084 and lies between the two phenotypes. **c** Tumors sensitive and insensitive to V-4084 were analyzed by microarray. We identified 301 differentially expressed genes (Student’s *t* test, *p* ≤ 0.005). While SF295 was not included in the analysis due to its partial sensitivity, it is in the heatmap between the *yellow lines*. Although clustering with the sensitive cell lines, SF295 tumors share similarities with the insensitive tumors

Because HGF-autocrine activation is the key molecular feature determining responsiveness to MET inhibitors, we asked whether sensitive glioma subcutaneous xenografts are transcriptionally similar to each other and are dissimilar from insensitive glioma models. We used microarrays to test sensitive (U87M2 and U118) and insensitive tumors (U251M2 and DBM2) treated for 7 days with either vehicle or V-4084 (Fig. 1d, *n* = 3). Unsupervised hierarchical clustering based on a whole-gene data set (33,304 transcripts, of which 22,372 were annotated, GSE64667) showed that all sensitive tumors naturally clustered together and were separated from the insensitive ones, indicating common genomic features among tumors driven by HGF (Fig. 2a). Looking further at the sensitive tumors (U87M2 and U118), we observed that V-4084 treatment did not change the expression profiles of the tumors; i.e., all U87M2 tumors regardless of treatment clustered together, distinct from U118 tumors. Furthermore, treated and untreated tumors within each glioma cell line xenograft clustered together, suggesting that the constitutive gene expression in these models was not vulnerable to events driven by signaling perturbation upstream (MET inhibition). In contrast, clustering pre- and post-treatment of DBM2 and U251M2 glioma lines was less tight between vehicle and treated tumors indicating that MET inhibition had a global effect on gene expression profiles of these models (Fig. 2a). Principal Component Analysis (PCA, Fig. 2b), which identifies gene expression patterns (principal components) that explain the variance across a data set, revealed that all sensitive tumors were closer to each other and further from the insensitive tumors, regardless of V-4084 treatment. The SF295 model showed partial sensitivity to V-4084, and its transcriptional profile was shown to be intermediate between those of the sensitive and insensitive lines (Fig. 2a).

To narrow the roster of genes most associated with HGF-autocrine activation in the xenograft studies, we analyzed the transcriptional profiles of sensitive (U87M2 and U118) and insensitive tumors (DBM2 and U251M2) without V-4084 treatment and identified 301 genes that were differentially expressed between the two groups (Fig. 2c; Student’s *t* test, p ≤ 0.005). While SF295 was not included in the initial analysis due to its partial sensitivity to V-4084, its expression data is included in the heatmap (Fig. 2c, between the yellow lines). We show that sensitive and insensitive tumors were discretely separable from each other. Moreover, although SF295 statistically clustered with the sensitive cell lines, it also showed similarities to the insensitive lines (Fig. 2c). Among the 301 genes, the most up-regulated gene was HGF, supporting its role as a driver of the sensitive phenotype. As we applied ingenuity pathway analysis (IPA) to depict potential pathways populated by the 301 genes, we found that “Glioma Invasiveness Signaling” was the third best-fit pathway based on the differentially-expressed genes between sensitive and insensitive glioma cell lines (Additional file 1: Fig. S2), supporting that HGF-autocrine activation is a strong molecular feature that drives GBM invasiveness.

### A Molecular signature indicating GBM responsiveness to MET inhibitors

**Fig. 3:**
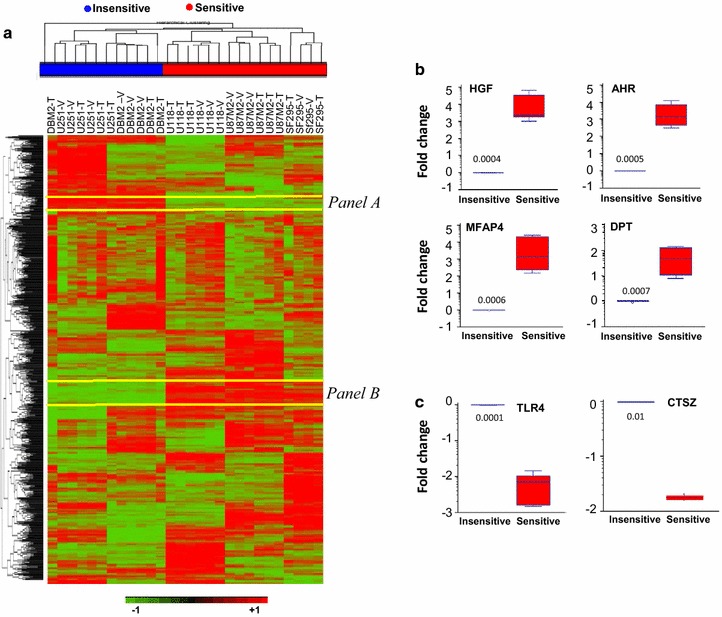
An HGF signature separates sensitive and insensitive models. **a** Using the TCGA data sets and approach [14], the transcriptional profiles of patients having high or low HGF expression were compared, and 887 genes were identified as differentially expressed (Student’s *t* test, *p* ≤ 0.0001). After clustering these genes with the glioma cell line xenograft data sets, we found 56 genes that were uniquely down- (*Panel A*) or up-regulated (*Panel B*) in the sensitive tumors. Among them there are 21 genes overlapping with those found in Fig. 2C, providing a signature of an HGF network (Table 1) that may identify tumors sensitive to MET inhibitors. **b**–**c** From the 21 gene signature, selected genes that are up-regulated (**b**) or down-regulated (**c**) were validated using qPCR. mRNAs from U87M2 and U118 were used for sensitive tumors, and mRNAs from U251M2 and DBM2 were used for insensitive tumors. Fold change = log (signal intensity from sensitive tumors/signal intensity from insensitive tumors)

Our earlier analysis of TCGA data showed that approximately 30 % of GBMs display overexpression of HGF and MET, suggesting instances in the patient population where autocrine HGF activation occurs [14]. Using the same criteria as we reported previously [14], which posited the top 10 % of GBM specimens with the highest HGF expression as tumors with HGF-autocrine activation, we contrasted the transcriptional profiles of tumors having high and low HGF expression. We found 887 differentially expressed genes in GBM patients with high HGF expression (Student’s *t* test, p ≤ 0.00001). When clustering the 887 genes using the glioma cell line xenograft tumor data sets, we observed that  out of 887 genes only 56 were able to clearly separate sensitive (U87M2 and U118) and insensitive (DBM2 and U251M2) tumors (Fig. 3a, panels A and B). Interestingly, 21 out of 56 (37.5 %) were included in the 301-gene profile (Table 1), providing a promising signature that may predict whether or not GBM patients will respond to MET inhibitors. The most differentially expressed genes (TLR4 and CTSZ in Panel A; HGF, AHR, MFAP4, and DPT in Panel B, Table 1) were validated by quantitative real-time PCR (qPCR) in xenograft tumors, showing concordance to microarray data (Fig. 3b). That all up- or down-regulated genes are tightly clustered together in their own groups suggests a biological relevance among these genes. Our results suggest that the overexpression of HGF is associated with a functional network through which sensitivity to MET inhibitors is determined.

**Table 1:** The HGF signature genes

GeneSymbol	GeneName	Ratio^a^	P value	Chromosome
From panel A: genes that are down-regulated only in sensitive tumors (*n* = 9)				
GPLD1	Glycosylphosphatidylinositol specific phospholipase D1	0.48	0.0019391	14
NOVA2	Neuro-oncological ventral antigen 2	0.46	0.0026136	7
LRP5	Low density lipoprotein receptor-related protein 5	0.41	0.0028902	11
ARHGEF4	Rho guanine nucleotide exchange factor (GEF) 4	0.35	0.0013269	2
F11R	F11 receptor	0.33	0.0005396	1
ALDH5A1	Aldehyde dehydrogenase 5 family, member A1	0.31	0.0003027	6
SLIT3	Slit homolog 3 (Drosophila)	0.22	0.000271	11
TLR4	Toll-like receptor 4	0.088	2.59E−05	9
CTSZ	Cathepsin Z	0.081	0.0044628	20
From panel B: genes that are up-regulated only in sensitive tumors (*n* = 12)				
HGF	Hepatocyte growth factor (hepapoietin A; scatter factor)	47.52	0.000534	7
AHR	Aryl hydrocarbon receptor	46.56	2.35E−05	7
MFAP4	Microfibrillar-associated protein 4	28.51	8.53E−05	17
DPT	Diptericin	9.37	0.0027492	2R
COL3A1	Collagen, type III, alpha 1	8.210	0.0012553	2
F2RL2	Coagulation factor II (thrombin) receptor-like 2	3.96	0.003144	5
LPXN	Leupaxin	3.86	0.0038993	11
DAB2	Dab, mitogen-responsive phosphoprotein, homolog 2	3.5	0.0035502	5
TBC1D8B	TBC1 domain family, member 8B (with GRAM domain)	2.7	0.0015932	X
GPHN	Gephyrin	2.63	0.0038916	6
C16orf45	Chromosome 16 open reading frame 45	1.99	0.0048897	16
CREB3L2	cAMP responsive element binding protein 3-like 2	1.87	0.0047229	7

By analyzing the mRNA expression datasets from TCGA GBM patients and those from preclinical xenograft models, 21 genes were found uniquely down- or up-regulated only in the sensitive tumors, providing a signature of an HGF network to identify tumors sensitive to MET inhibitors

^a^Ratio = average mRNA expression level in insensitive tumors/average mRNA expression level in sensitive tumors

### The HGF signature identifies sensitivity to MET inhibitors in GBM PDX models

**Fig. 4:**
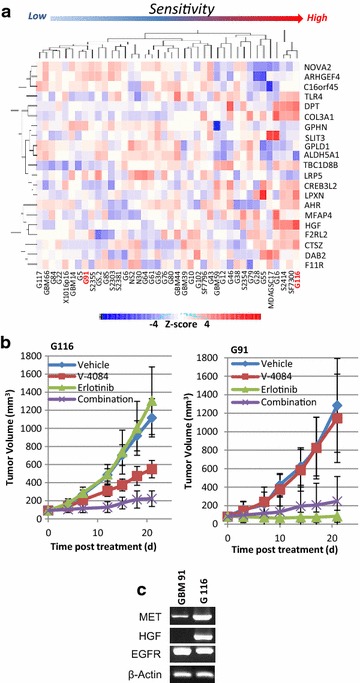
HGF signature ranks the predicted sensitivity of GBM-PDX models to MET inhibitors. **a** Forty GBM patient-derived tumor models were analyzed by using the HGF signature as a biomarker of suitability for treatment with MET inhibitors. Models with high HGF expression are clustered together at right side of the heatmap, suggesting a common molecular profile and a high sensitivity to MET-targeted therapy. **b** GBM models G116 and G91 were used to validate the therapeutic efficacy of V-4084. G116, ranked the most sensitive to MET inhibitor, showed significant response to V-4084 treatment; G91, ranked the insensitive, showed no response. **c** HGF, MET, and EGFR mRNA expression levels in G116 and G91 tumor models

To further evaluate the HGF signature’s predictive ability, a set of 40 GBM patient-derived xenograft models with matched genomic profiles generated by the Ivy GBM Consortium (GSE39242) was used for validation analysis. Using the 21-gene signature, we clustered the Ivy GBM Consortium models according to predicted sensitivity to MET inhibition (Fig. 4a). While the models with the highest HGF expression level were naturally clustered to one end, those with low or no HGF expression levels were clustered to the other end. To validate the signature’s predictive ability, G116, and G91 which showed highest or no HGF expression levels (Fig. 4c) were tested for sensitivity to V-4084, erlotinib and the combination of the two (Fig. 4b). We found that G116 was highly sensitive to V-4084 alone, but erlotinib had no effect, while G91 showed exactly the opposite. These results suggest a mutually exclusive effect by the two RTKs and support the previous finding that MET negatively correlates with EGFR expression in primary GBM.

### Host-tumor interaction in response to MET kinase inhibitor

Although it is well accepted that the host’s microenvironment regulates tumor growth, genomic approaches have not been used to dissect host/tumor cross talk or to delve into ways targeted therapy alters the host (non-tumor) cells. To explore this, we combined the use of human and mouse microarrays to study gene expression changes in tumor cells and host cells in response to MET inhibitors. mRNA samples from pre- and post-treatment tumors were used in transcriptional profile analysis on both human and mouse microarrays. The molecular pathway data from the human microarray portrays the tumor response to V-4084 treatment (Additional file 1: Fig. S3), and the data from the mouse microarray represent the microenvironmental response (Additional file 1: Fig. S4). From the human array data sets, we identified 485 genes that were differentially expressed in treated tumors (Student’s *t* test, treated vs. vehicle, p ≤ 0.01). A supervised cluster based on the 485 genes showed a clear separation between treated and vehicle samples only in U87M2 and U118 tumors, which have HGF-autocrine activation (Additional file 1: Fig. S3A, B). In contrast, none of the insensitive tumors showed clear separation, consistent with treatment having little effect (Additional file 1: Fig. S3A). To plot the most significant genes (n = 550, Student’s *t* test, treated vs. vehicle, p ≤ 0.01) and signaling pathways affected by V-4084 treatment, we performed the same analysis using only the two most sensitive tumor models, U87M2 and U118, and found that the 10 most affected signaling pathways were almost all associated with cell cycle regulation (Additional file 1: Fig. S3C), which is consistent with other groups’ reports that the MET kinase inhibitor SGX523 impedes cancer cell proliferation and cell cycle progression [19, 20].

In order to study the host response to MET kinase inhibitors, the same mRNA samples used for the human microarray were analyzed by Affymetrix mouse microarrays. Consistent with the observation from the human array (Fig. 3a), unsupervised clustering and PCA analysis performed on the whole mouse gene data set (*n* = 25,255 transcripts) showed a clear separation between sensitive and insensitive tumors (Additional file 1: Fig. S4A, B). Analyzing treated vs. vehicle tumors using mouse data sets for only U87M2 and U118 tumors revealed 370 genes that were differentially expressed (Student’s *t* test, p ≤ 0.01). Interestingly, the most highly altered signaling pathway in the host (Cell cycle: G2/M DNA Damage Checkpoint regulation) turned out to be cell-cycle-regulation related. Altogether, four pathways (Mitotic Roles of Polo-Like Kinase; ATM Signaling; Cell Cycle: G2/M DNA Damage Checkpoint Regulation; and Estrogen-Mediated S-Phase Entry) were the same as from the human array results which indicate the response from the tumor side (Additional file 1: Fig. S3C). To eliminate the possibility that a pathway identified from both human and mouse arrays might come from the overlapping design of the array probes, we carefully compared the differentially expressed genes from both arrays and found no redundancies (Additional file 1: Fig. S4E). Our data suggest that although MET inhibitors have consequences on distinct molecular processes on subcutaneous glioma tumor cells and host cells within the tumor (endothelial cells, macrophages, stromal elements, etc.), V-4084 affects cell cycle in both tumor and host.

### MET activation in EGFR^*amp*^ GBM resistant to Erlotinib

**Fig. 5:**
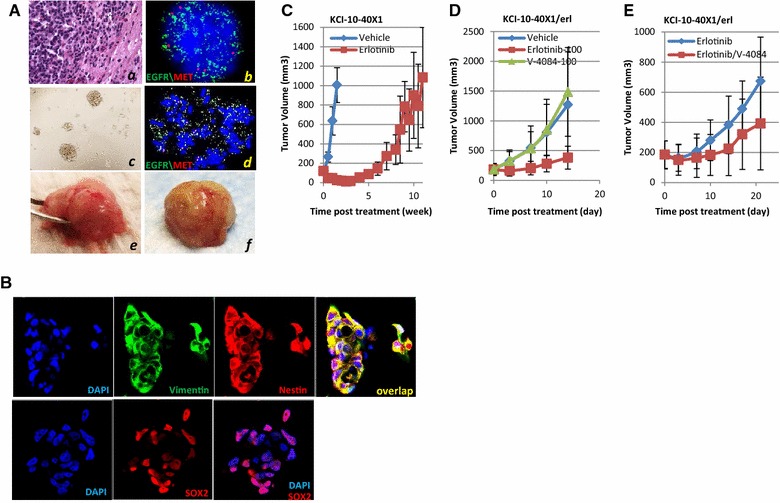
Therapeutic efficacy of MET and EGFR inhibitors using the KCI-10-40 PDX model. **A** Characterization of the KCI-10-40X1 PDX model. KCI-10-40 primary GBM showed 29.5 % EGFR amplification (*a*, *b*). After the primary tumor was grown as a xenograft, neurosphere cells were derived for in vitro growth (*c*) which showed 100 % EGFR amplification (*d*). These cells induce intracranial tumor growth in the mouse brain (*e* and *f*). **B** KCI-10-40X1 cells express nestin, vimentin, and Sox2, as shown by immunofluorescence staining, indicating stem-cell-like properties. **C** KCI-10-40X1 tumors showed high sensitivity to erlotinib treatment (75 mg/kg); tumor regression could be seen after 1 week. However, after 5 weeks of continuous treatment at 100 mg/kg, tumors began to regrow much faster, indicating the start of acquired resistance. Note that KCI-10-40X1 took 2 weeks to reach 1000 mm^3^ in size, while the resistant tumor (KCI-10-40X1/erl) took almost 11 weeks to reach similar size. **D** Although KCI-10-40X1/erl tumors grow while on erlotinib treatment, discontinuing treatment (vehicle) accelerated tumor growth. V-4084 (100 mg/kg) alone did not inhibit KCI-10-40X1/erl tumor growth. **E** V-4084 (100 mg/kg) in combination with erlotinib (100 mg/kg) inhibited KCI-10-40X1/erl tumor growth

Although EGFR^*amp*^ occurs in about 45 % of GBM patients, clinical trials using EGFR inhibitors failed to show activity. To test whether MET pathway activation may serve as a bypass mechanism, [8] we established a patient-derived EGFR^*amp*^ GBM model (KCI-10-40) with acquired resistance to erlotinib, then measured its sensitivity to MET inhibitor (Fig. 5). While 29.5 % of the cells in the primary tumor carry EGFR^*amp*^ (Fig. 5A, *a*, *b*), isolated neurosphere cells showed 100 % EGFR^*amp*^ (Fig. 5A, *c*, *d*). These cells express nestin, vimentin, and SOX2 (Fig. 5B) and show malignant orthotopic tumor growth (Fig. 5A, *e*, *f*), indicating that EGFR^*amp*^ is serially-maintained in the glioma stem-cell-like subpopulation.

To induce acquired resistance, KCI-10-40X1cells were inoculated into nude mice subcutaneously followed by continuous erlotinib treatment (75–100 mg/kg). While significant tumor regression was observed in the first week (Fig. 5C), tumors started to re-grow after 5 weeks of continuous treatment, with progressively increasing growth rate, consistent with the manifestation of a rescue pathway independent of EGFR. The same transplant procedure was serially repeated four times to establish the tumor model adaptive to erlotinib treatment (KCI-10-40X1/erl). In vivo, simply switching treatment from erlotinib to V-4084 did not inhibit tumor growth (Fig. 5D). A combination of V-4084 and erlotinib, however, retarded KCI-10-40X1/erl tumor growth (Fig. 5E). Concordant with our previous results demonstrating that inhibition of the MET pathway in U87 tumors result in EGFR pathway activation [14], this study support a biological reciprocity between the two pathways and provides additional evidence for the combined use of MET and EGFR inhibitors in treating GBM patients with EGFR^*amp*^.

## Discussion

Standard-of-care for treating GBM involves maximum surgical resection followed by the Stupp regimen consisting of fractionated radiotherapy plus concurrent daily chemotherapy using the alkylating agent TMZ, and 6-12 cycles of adjuvant TMZ [21]. However, in spite of this aggressive multimodal approach, local invasion and tumor recurrence is seen in nearly all patients and is largely due to the highly infiltrative and adaptive GBM cells [22]; and, overall, the median patient survival remains a dismal at less than 15 months with a 5-year survival rate less than 5 %. As such, there has been considerable interest in recent years in applying a targeted approach to GBM patients. The success of targeted therapies depends on both knowledge of the essential molecular features that drive pathway activity and proper selection of the patient population likely to respond favorably to the specific treatment. Examples of such successes include the use of EGFR^*T790M*^ as a marker for erlotinib treatment of non-small-cell lung cancer (NSCLC) patients [23] and use of BRAF^*V600E*^ for vemurafenib treatment of melanoma patients [24]. Our prior studies have shown that MET inhibitors can effectively impair HGF-autocrine GBM tumor growth [14, 25]. In this study, we further demonstrated that HGF-autocrine-driven GBM invasion can be significantly blocked by MET inhibitors (Fig. 1); these findings support the use of HGF-autocrine activation as a biomarker for identifying GBM patients most likely to benefit from treatment with MET inhibitors. This result raises the prospect for a potential clinical application in GBM patients with HGF-autocrine activation, where use of MET inhibitors before surgical resection may target the invasive tumor cells at the leading edge and help to better define the tumor margin and facilitate maximal surgical removal. Likewise, treating GBM patients with MET inhibitors after surgical debulking may enhance the efficacy of adjuvant radiation and chemotherapy by arresting the invading glioma cells.

Despite the large collection of primary tumor data sets that TCGA has generated, this profiling data proves of limited direct use when the aim is to discover predictive signatures for response to specific treatments. Presently, very few patients enrolled in clinical trials of targeted therapeutics undergo systematic profiling of their GBM tissue in an attempt to align unique genomic signatures with response to the targeted drug. Advancement of new targeted agents, i.e., chemical probes, could be facilitated by more parallel study of panels of relevant preclinical models that are genomically profiled. To date, only hypermethylation of the O6-methylguanine-DNA-methyltransferase (MGMT) gene, which has been shown to be a predictive marker of sensitivity to alkylating agents (such as TMZ) and associated with improved outcome, has been routinely employed in a clinical setting as a predictive signature in GBM patients [13]. In contrast, therapeutic efficacy using xenograft models is easy to determine, however, concerns remain regarding how closely xenograft models resemble human cancer biology.

In this study, we developed a two-step strategy to identify tumors that are sensitive to MET-inhibiting drugs and to identify the genes that were highly associated with HGF overexpression and that were up- or down-regulated coincident to MET-inhibition response. We first conducted a training analysis with TCGA data sets to identify up- or down-regulated genes in GBM tumors which overexpressed HGF. A data mingling using TCGA human data together with analysis of the xenograft database eliminated the “non-human” factors from the xenograft model data sets. Although 887 and 301 genes were differentially expressed in the human and xenograft data sets, a subset of 21 genes was able to clearly separate responders from nonresponders, demonstrating the value of using human data sets to help inform the results from xenograft studies. In the next step, a data set independently derived from GBM PDX orthotopic models was used for validation of predictive therapeutic efficacy. The heatmap showed a cluster of models highly correlated to HGF expression, but it also showed that other components were involved in determining vulnerability to MET inhibition. The 21-gene signature may represent a functional HGF network, although a biological inference towards a hallmark or a phenotype requires further study. Most importantly, after therapeutic validation, the prediction of G116 as a responder and G91 as a non-responder was accurate (Fig. 4), highlighting the potential of this signature for enrolling patients in MET-targeted therapy. Although extensive validation (i.e., through repeating step two) is needed to optimize the molecular signature for clinical purposes, our study is a “proof-of-concept” that combining TCGA primary tumor datasets (human) and xenograft tumor model datasets (human tumor grown in mice) using therapeutic efficacy as an endpoint may serve as a useful approach to discover and develop molecular signatures as therapeutic biomarkers for targeted therapy.

Although genomic and proteomic tools have been widely used to analyze GBM subtypes [5, 6], to map out specific mutations and signaling pathways [4], or to identify therapeutic targets related in particular to MET and EGFR*vIII* in combination [11], these approaches have not been used to interpret micro-environmental regulation. The result of using human and mouse arrays to identify the core pathways affected by MET inhibitors in the context of tumor/host crosstalk is speculative but very promising. Although the use of human xenograft tumor models can be debated due to the loss of human host cell biology, in our study, the use of specific human and mouse arrays allows us to measure the signaling pathways impacted in the host and tumor compartments, by which the biological response from host and tumor can be viewed independently. As we have shown, the genes differentially expressed from the human array (*n* = 550) are very different from those in the mouse array (*n* = 370), with no overlapping genes. Although nude mice are claimed not to have an intact immune system, we observed pathways such as host-versus-graft disease signaling and antigen presentation to be up-regulated in sensitive xenografts, indicating an increased immune reaction in the host that might be required for treatment efficacy [26]. On the tumor side, all pathways identified were associated with cell-cycle regulation. Strikingly, non-overlapping genes from the tumor and the host still yielded overlapping pathways, with cell-cycle regulation as the common process. Our study, then, uses xenograft mouse models plus human and mouse arrays to provide preliminary, yet important, information about the tumor/host interaction in response to MET inhibitors. Although a more clinically-relevant analysis of tumor/host crosstalk requires the use of orthotopic models, we suggest that for GBM patients in clinical trials, the immune reactions of individual patients might help identify vulnerability to MET inhibitors.

A number of RTK inhibitors have entered cancer clinical trials with limited efficacy; one of the major obstacles noted has been the rapid development of acquired resistance to the targeted drug [27, 28]. MET pathway activation has been frequently reported as a mechanism of tumor recurrence in NSCLC (EGFR^*T790M*^) treated with erlotinib [23, 29], in melanoma (BRAF^*V600E*^) treated with vemurafenib [24], and in GBM treated with bevazicumab [30]. Preclinically, MET inhibitors have been used to induce resistance via different mechanisms in different cancer types [31]. While findings repeatedly emphasize the importance of targeting the MET pathway in primary and recurrent cancer, the strategies are shifting from monotherapy to multi-target therapy. Although EGFR^*amp*^ is one of the most common genetic alterations in GBM and is often accompanied by constitutively elevated p-EGFR, clinical trials using EGFR inhibitors such as erlotinib or gefitinib have invariably failed to provide clinically meaningful benefit to patients harboring a GBM. The mechanisms leading to such failures include dynamic regulation of extrachromosomal mutant EGFR DNA [32], up-regulation of PI3Kp110δ [33], and depression of PDGFRβ transcription [34]. Previously, we observed that expression of MET correlated negatively with EGFR and that long-term exposure to MET inhibitors in the U87MG model induced resistance via the EGFR pathway. This observation indicated an intrinsic balance between MET and EGFR, i.e., inhibiting one may activate the other. Here, we further tested whether inhibiting EGFR causes MET activation as a rescue pathway and whether a combination of the two RTK inhibitors would improve the efficacy in treating EGFR^*amp*^ GBM that escape erlotinib treatment. By using KCI-10-40X1, a PDX model derived from a GBM patient with EGFR^*amp*^, we found that a combination of V-4084 and erlotinib inhibited KCI-10-X1/erl-res tumor growth and provide additional evidence to treat GBM EGFR^*amp*^ patients targeting both EGFR and MET. The mechanisms underlying how the MET-EGFR interaction controls drug sensitivity require further study.

## Conclusion

In summary, specific MET inhibitors block HGF-autocrine-dependent GBM proliferation and invasion. Using HGF-autocrine activation as a biomarker, we developed a molecular signature that may be used to predict sensitivity to MET inhibitors. The MET inhibitors regulate tumor and host crosstalk, and overall impede tumor growth by inhibiting cell cycle progression. We also suggest that long-term exposure of EGFR^*amp*^ GBM to erlotinib treatment may initiate MET pathway activation, further supporting the earlier use of MET and EGFR inhibitors in combination for treating malignant GBM.

## Additional file

**Additional file 1:** Supplementary data including Supplementary Methods, 1 Supplementary Table, 4 Supplementary Figures and Supplementary Reference.

## Abbreviations

GBM: glioblastoma
HGF: hepatocyte growth factor
PDX: models patient derived xenograft models
TCGA: the Cancer Genome Atlas Network
FISH: fluorescence in situ hybridization
TMZ: temozolomide
PCA: principle Component Analysis
IPA: ingenuity Pathway Analysis
NSCLC: non small cell lung cancer
MGMT: O6-methylguanine-DNA-methyltransferase

## References

[1] Giese A, Bjerkvig R, Berens ME, Westphal M (2003). Cost of migration: invasion of malignant gliomas and implications for treatment. J Clin Oncol.

[2] Rao JS (2003). Molecular mechanisms of glioma invasiveness: the role of proteases. Nat Rev Cancer.

[3] Birchmeier C, Birchmeier W, Gherardi E, Vande Woude GF (2003). Met, metastasis, motility and more. Nat Rev Mol Cell Biol.

[4] Network CGAR (2008). Comprehensive genomic characterization defines human glioblastoma genes and core pathways. Nature.

[5] Phillips HS, Kharbanda S, Chen R, Forrest WF, Soriano RH, Wu TD, Misra A, Nigro JM, Colman H, Soroceanu L (2006). Molecular subclasses of high-grade glioma predict prognosis, delineate a pattern of disease progression, and resemble stages in neurogenesis. Cancer Cell.

[6] Verhaak RG, Hoadley KA, Purdom E, Wang V, Qi Y, Wilkerson MD, Miller CR, Ding L, Golub T, Mesirov JP (2010). Integrated genomic analysis identifies clinically relevant subtypes of glioblastoma characterized by abnormalities in PDGFRA, IDH1, EGFR, and NF1. Cancer Cell.

[7] Addeo R, Zappavigna S, Parlato C, Caraglia M (2014). Erlotinib: early clinical development in brain cancer. Expert Opin Investig Drugs.

[8] Raizer JJ, Abrey LE, Lassman AB, Chang SM, Lamborn KR, Kuhn JG, Yung WK, Gilbert MR, Aldape KD, Wen PY (2010). A phase I trial of erlotinib in patients with nonprogressive glioblastoma multiforme postradiation therapy, and recurrent malignant gliomas and meningiomas. Neuro Oncol.

[9] Chakravarti A, Wang M, Robins HI, Lautenschlaeger T, Curran WJ, Brachman DG, Schultz CJ, Choucair A, Dolled-Filhart M, Christiansen J (2013). RTOG 0211: a phase 1/2 study of radiation therapy with concurrent gefitinib for newly diagnosed glioblastoma patients. Int J Radiat Oncol Biol Phys.

[10] Lassman AB, Rossi MR, Raizer JJ, Abrey LE, Lieberman FS, Grefe CN, Lamborn K, Pao W, Shih AH, Kuhn JG (2005). Molecular study of malignant gliomas treated with epidermal growth factor receptor inhibitors: tissue analysis from North American Brain Tumor Consortium Trials 01-03 and 00-01. Clin Cancer Res.

[11] Huang PH, Mukasa A, Bonavia R, Flynn RA, Brewer ZE, Cavenee WK, Furnari FB, White FM (2007). Quantitative analysis of EGFRvIII cellular signaling networks reveals a combinatorial therapeutic strategy for glioblastoma. Proc Natl Acad Sci USA.

[12] Lal B, Goodwin CR, Sang Y, Foss CA, Cornet K, Muzamil S, Pomper MG, Kim J, Laterra J (2009). EGFRvIII and c-Met pathway inhibitors synergize against PTEN-null/EGFRvIII + glioblastoma xenografts. Mol Cancer Ther.

[13] Brennan CW, Verhaak RG, McKenna A, Campos B, Noushmehr H, Salama SR, Zheng S, Chakravarty D, Sanborn JZ, Berman SH (2013). The somatic genomic landscape of glioblastoma. Cell.

[14] Xie Q, Bradley R, Kang L, Koeman J, Ascierto ML, Worschech A, De Giorgi V, Wang E, Kefene L, Su Y (2012). Hepatocyte growth factor (HGF) autocrine activation predicts sensitivity to MET inhibition in glioblastoma. Proc Natl Acad Sci USA.

[15] Garber K (2014). MET inhibitors start on road to recovery. Nat Rev Drug Discov.

[16] Xie Q, Thompson R, Hardy K, DeCamp L, Berghuis B, Sigler R, Knudsen B, Cottingham S, Zhao P, Dykema K (2008). A highly invasive human glioblastoma pre-clinical model for testing therapeutics. J Transl Med.

[17] Gentleman RC, Carey VJ, Bates DM, Bolstad B, Dettling M, Dudoit S, Ellis B, Gautier L, Ge Y, Gentry J (2004). Bioconductor: open software development for computational biology and bioinformatics. Genome Biol.

[18] Livak KJ, Schmittgen TD (2001). Analysis of relative gene expression data using real-time quantitative PCR and the 2(-Delta Delta C(T)) Method. Methods.

[19] Guessous F, Zhang Y, diPierro C, Marcinkiewicz L, Sarkaria J, Schiff D, Buchanan S, Abounader R (2010). An orally bioavailable c-Met kinase inhibitor potently inhibits brain tumor malignancy and growth. Anticancer Agents Med Chem.

[20] Zhang YW, Staal B, Essenburg C, Su Y, Kang L, West R, Kaufman D, Dekoning T, Eagleson B, Buchanan SG (2010). MET kinase inhibitor SGX523 synergizes with epidermal growth factor receptor inhibitor erlotinib in a hepatocyte growth factor-dependent fashion to suppress carcinoma growth. Cancer Res.

[21] Neagu MR, Huang RY, Reardon DA, Wen PY (2015). How treatment monitoring is influencing treatment decisions in glioblastomas. Curr Treat Options Neurol.

[22] Xie Q, Mittal S, Berens ME (2014). Targeting adaptive glioblastoma: an overview of proliferation and invasion. Neuro Oncol.

[23] Turke AB, Zejnullahu K, Wu YL, Song Y, Dias-Santagata D, Lifshits E, Toschi L, Rogers A, Mok T, Sequist L (2010). Preexistence and clonal selection of MET amplification in EGFR mutant NSCLC. Cancer Cell.

[24] Straussman R, Morikawa T, Shee K, Barzily-Rokni M, Qian ZR, Du J, Davis A, Mongare MM, Gould J, Frederick DT (2012). Tumour micro-environment elicits innate resistance to RAF inhibitors through HGF secretion. Nature.

[25] Xie Q, Su YL, Dykema K, Johnson J, Koeman J, De Giorgi V, Huang A, Schlegel R, Essenburg C, Kang L (2013). Overexpression of HGF promotes HBV- incuded hepatocellular carcinoma progression and is an effective indicator for Met-targeting therapy. Genes Cancer.

[26] Ascierto ML, Kmieciak M, Idowu MO, Manjili R, Zhao Y, Grimes M, Dumur C, Wang E, Ramakrishnan V, Wang XY (2012). A signature of immune function genes associated with recurrence-free survival in breast cancer patients. Breast Cancer Res Treat.

[27] Gherardi E, Birchmeier W, Birchmeier C, Woude GV (2012). Targeting MET in cancer: rationale and progress. Nat Rev Cancer.

[28] Xie Q, Vande Woude GF, Berens ME (2012). RTK inhibition: looking for the right pathways toward a miracle. Future Oncol.

[29] Bean J, Brennan C, Shih JY, Riely G, Viale A, Wang L, Chitale D, Motoi N, Szoke J, Broderick S (2007). MET amplification occurs with or without T790M mutations in EGFR mutant lung tumors with acquired resistance to gefitinib or erlotinib. Proc Natl Acad Sci USA.

[30] Lu KV, Chang JP, Parachoniak CA, Pandika MM, Aghi MK, Meyronet D, Isachenko N, Fouse SD, Phillips JJ, Cheresh DA (2012). VEGF inhibits tumor cell invasion and mesenchymal transition through a MET/VEGFR2 complex. Cancer Cell.

[31] Qi J, McTigue MA, Rogers A, Lifshits E, Christensen JG, Janne PA, Engelman JA (2011). Multiple mutations and bypass mechanisms can contribute to development of acquired resistance to MET inhibitors. Cancer Res.

[32] Nathanson DA, Gini B, Mottahedeh J, Visnyei K, Koga T, Gomez G, Eskin A, Hwang K, Wang J, Masui K (2014). Targeted therapy resistance mediated by dynamic regulation of extrachromosomal mutant EGFR DNA. Science.

[33] Schulte A, Liffers K, Kathagen A, Riethdorf S, Zapf S, Merlo A, Kolbe K, Westphal M, Lamszus K (2013). Erlotinib resistance in EGFR-amplified glioblastoma cells is associated with upregulation of EGFRvIII and PI3Kp110delta. Neuro Oncol.

[34] Akhavan D, Pourzia AL, Nourian AA, Williams KJ, Nathanson D, Babic I, Villa GR, Tanaka K, Nael A, Yang H (2013). De-repression of PDGFRbeta transcription promotes acquired resistance to EGFR tyrosine kinase inhibitors in glioblastoma patients. Cancer Discov.

