# Supplementary material for: Genomic profiling of a Hepatocyte growth factor-dependent signature for MET-targeted therapy in glioblastoma
Source: J Transl Med. 2015 Sep 17;13:306. doi: 10.1186/s12967-015-0667-x (PMC4574608; doi:10.1186/s12967-015-0667-x)
Supplement: Supplementary file 1 — Additional file 1: Supplementary data including Supplementary Methods, 1 Supplementary Table, 4 Supplementary Figures and Supplementary Reference. [file 12967_2015_667_MOESM1_ESM.doc]

**Additional File 1**

**Genomic profiling of a predictive signature for MET-targeted therapy in glioblastoma**

**Running Title:** A molecular signature for MET-targeted therapy

Jennifer Johnson,^1^ Maria Libera Ascierto,^2,3^ Sandeep Mittal,^4^ David Newsome,^5^ Liang Kang,^1^ Michael Briggs,^6^ Kirk Tanner,^5^ Michael E. Berens,^7^ Francesco M. Marincola,^2,8^ George F. Vande Woude,^1^ and Qian Xie^1*^

^1^Center for Cancer and Cell Biology, Van Andel Research Institute, Grand Rapids, MI, USA
^2^Department of Transfusion Medicine, Infectious Disease and Immunogenetics Section, Clinical Center, and Trans-National Institutes of Health Center for Human Immunology, National Institutes of Health, Bethesda, MD, USA

^3^Department of Oncology, Johns Hopkins University, Baltimore, MD, USA
^4^Departments of Neurosurgery and Oncology, Karmanos Cancer Institute, Wayne State University, Detroit, MI, USA
^5^Vertex Pharmaceutical Inc., Boston, MA, USA
^6^Woodland Pharmaceuticals, Shrewsbury, MA, USA
^7^Translational Genomics Research Institute, Phoenix, AZ, USA

^8^Research Branch, Sidra Medical and Research Center, Doha, Qatar

**Files in this Data Supplement:**

Supplementary Methods.

Supplementary Table 1. Selectivity of V-4084 against kinase panel.

Supplementary Figures 1-4. Supplementary Figure 1. V-4084 inhibited HGF-paracrine-activated proliferation and invasion. Supplementary Figure 2. Top ten affected signaling pathways activated in HGF-autocrine tumors that are sensitive to MET inhibitors. Supplementary Figure 3. Gene expression changes in glioma xenograft tumors in response to V-4084 treatment determined by human array.

Supplementary Figure 4. Gene expression changes in host cells within glioma cell line xenografts in response to V-4084 treatment determined by mouse array.

Supplementary Reference

**Supplementary Methods**

**Kinase inhibitory assay**

The inhibitory activity of V-4084 against MET kinase was determined by following the residual kinase activity of MET using a radiometric assay. The final concentration of the components in the assay were as follows: 100 mM HEPES (pH 7.5), 10 mM MgCl_2_, 1 mM DTT, 0.01% BSA, 0.5 nM MET, 0.5 mg/ml polyE4Y, and 35 mM ^33^P-g-ATP. A stock solution of VRT-0846198 was made up in DMSO, from which additional dilutions were made in DMSO; a 1.5 mL aliquot of DMSO or inhibitor in DMSO was added to each well. Fifty microliters of a 2x substrate mixture (100 mM HEPES, 10 mM MgCl_2_, 1.0 mg/mL polyE4Y, and 70 mM ^33^P-g-ATP) was added and mixed with the inhibitor/DMSO. The reaction was initiated by the addition of 50 mL of a 2x enzyme mixture (100 mM HEPES (pH 7.5), 10 mM MgCl_2_, 2 mM DTT, 0.02% BSA, 1.0 nM MET. After 15 min, the reaction was quenched with 50 mL of 20% TCA. The quenched reaction was transferred to GF/B filter plates and washed 3 times with 5% TCA. Following the addition of Ultimate Gold™ scintillation agent (50 mL), the samples were counted in a Packard TopCount. The radioactivity trapped is a measure of the residual MET kinase activity. From the activity vs. concentration of VRT-0846198 titration curve, the K_i_ value was determined by fitting the data to an equation for competitive tight binding inhibition kinetics using Prism software, version 4.0, San Diego, CA, USA.

**Thymidine incorporation assays**

Cells were seeded into 96-well plates (2000 cells per well) in DMEM supplemented with 10% FBS for 24 h. The cells were starved in DMEM without FBS for 48 h, and further incubated with or without HGF for 12 h. Four hours before analysis, 1 μCi of [^3^H]thymidine (PerkinElmer) was added. Cells were washed with PBS and [^3^H]thymidine incorporation was measured by precipitation of whole cells in chilled 10% trichloroacetic acid, solubilization of precipitates with lysis buffer (0.02 M NaOH, 0.1% SDS), suspension in scintillation mixture (Packard Bioscience), and measurement in a liquid scintillation counter (Perkin Elmer) as described previously.

**HGF-induced urokinase plasminogen (uPA) activity**

Cells were grown overnight in DMEM/10% FBS. Drugs were dissolved in DMSO and were serially diluted from stock concentrations into DMEM/10% FBS medium and added to the appropriate wells. Immediately after drug or reagent addition, HGF/SF (60 ng/ml) was added to all wells (with the exception of wells used as controls to calculate basal growth and uPA-plasmin activity levels). Twenty-four hours after drug and HGF/SF addition, plates were processed for the determination of plasmin activity as described previously. Wells were washed twice with DMEM (Life Technologies, Inc.), and 200 µl of reaction buffer (50% [v/v] 0.05 units/ml plasminogen in DMEM, 40% [v/v] 50 mM Tris buffer [pH 8.2], and 10% [v/v] 3 mM Chromozyme PL [Boehringer Mannheim] in 100 mM glycine solution] was added to each well. The plates were then incubated at 37 °C with 5% CO_2_ for 4 h, at which time the absorbance was read on an automated spectrophotometric plate reader at the single wavelength of 405 nm.

**HGF-induced downstream signaling pathway**

Cells were seeded in 10-cm dishes and were grown until 80% confluent. After serum starvation overnight, cells were treated with (or without) SGX523 for 4 h with (or without) human HGF/SF or EGF (100 ng/ml) for 20 min at 37 °C. The cells were washed twice with ice-cold 1x PBS, and whole-cell lysates were prepared using RIPA buffer. The protein concentrations were determined by DC protein assay. Equal amounts of total protein (30 µg) from cell lysates were loaded on a 4-20% SDS-PAGE gel (Invitrogen), transferred to a polyvinylidene difluoride (PVDF) membrane (Invitrogen), and detected using an ECL Western Blotting Detection System (GE Healthcare). We used antibodies against human Met (clone 25H2,), phospho-Met (Y1234/1235), human EGFR (clone D38B1), phosphor-EGFR (Y1068), AKT, phospho-AKT (S473), p42/44 MAPK, phospho-p42/44 MAPK (T202/Y204) (all from Cell Signaling Technology); β-actin (clone AC-15, Abcam); and HGF (Clone 7-2, produced at Van Andel Research Institute). Secondary antibodies used were goat anti-rabbit IgG-HRP and goat anti-mouse IgG-HRP (Santa Cruz Biotechnology).

**Fluorescence *in situ* hybridization (FISH)**

FISH probes were prepared from the purified RP11-371D15 and RP11-34D10 at locus 4q25 spanning gene *EGF*; RP5-1091E12 and RP11-708P5 at locus 7p11.2 spanning gene *EGFR*; RP11-24F4 and RP11-24F4 at locus 7q21.11 spanning gene *HGF*; and RP11-163C9 and RP11-564A14 at locus 7q31.2 spanning gene *MET*, as described previously. Images were analyzed using FISH View EXPO v6.0 software (ASI), and at least 50-200 cells were scored for each sample.

**RT-PCR**

RT-PCR was performed as previously described . Primers used in RT-PCR reactions were human HGF forward: 5′-CAGCGTTGGGATTCTCAGTAT-3′, reverse: 5′-CCTATGTTTGTTCGTGTTGGA-3′; human MET forward: 5′-ACAGTGGCATGTCAACATCGCT-3′, reverse: 5′-GCTCGGTAGTCTACAGATTC-3′; β-actin forward: 5′-GGCGGCAACACCATGTACCCT-3′, reverse: 5′- AGGGGCCGGACTCGTCATACT-3′; human EGFR forward: 5′-CTTCTTGCAGCGATACACTGC-3′, reverse, 5′-ATGCTCCAATAAATTCACTGC-3′; human EGFR*vIII* forward*:* 5′-ATGCGACCCTCCGGGACG-3′, reverse: 5′-ATTCCGTTACACACTTTGCGGC-3′ designed to flank the deletion of exons 2 to 7. Reverse transcription was at 50 °C for 30 min, followed by enzyme inactivation and hot-start PCR at 95 °C for 15 min. Denaturation, annealing, and extension were done at 94 °C, 57 °C, and 72 °C, respectively, for 1 min each for a total of 25 cycles. The reaction was completed with an extension period at 70 °C for 10 min.

**Immunofluorescence Staining**

KCI-10-40X1 cells were grown in 6-cm dishes with glass bottom and fixed in 4% paraformaldehyde for 15 min. After washing using TBST, cells were stained with primary antibodies (Nestin at 1:50, Sigma; Vimentin at 1:100, DAKO; and SOX2 at 1:100, Sigma) overnight at 4^◦^C. After TBST wash for 3 time, cells were incubated with secondary antibodies (Alexa fluo 488 goat anti-mouse or Alexa fluo 546 goat anti-rabbit antibodies, 1:100, Invitrogene) at room temperature for 2 hours. Imagines were taken under Zeiss model 510 confocal microscope.

**Supplementary Table 1.** **Selectivity of V-4084 against kinase panel**

| **Kinase** | **K_i_ (µM)** | **Kinase** | **K_i_ (µM)** |
| --- | --- | --- | --- |
| MET | 0.025 | PI3Kα | > 4 |
| CDK2 | > 4 | PI3Kγ | > 4 |
| FLT3 | > 4 | PKA | > 4 |
| GSK3β | > 4 | PLK1 | > 4 |
| IRAK4 | > 4 | ROCK1 | > 4 |
| JAK2 | > 4 | SRC1 | > 4 |
| JNK3 | > 4 | SYK | > 4 |
| KDR | > 4 | RON | > 4 |

A radiometric assay was performed to determine the inhibitory activity of V-4084 against MET and 15 additional protein kinases. The K*i* value was analyzed by using the equation for competitive tight binding inhibition kinetics (Prism, version 4.0, San Diego, CA, USA). Results show that V-4084 selectively inhibits the MET kinase activity with a K_i_ of 0.025 µM.

**Supplementary Figures
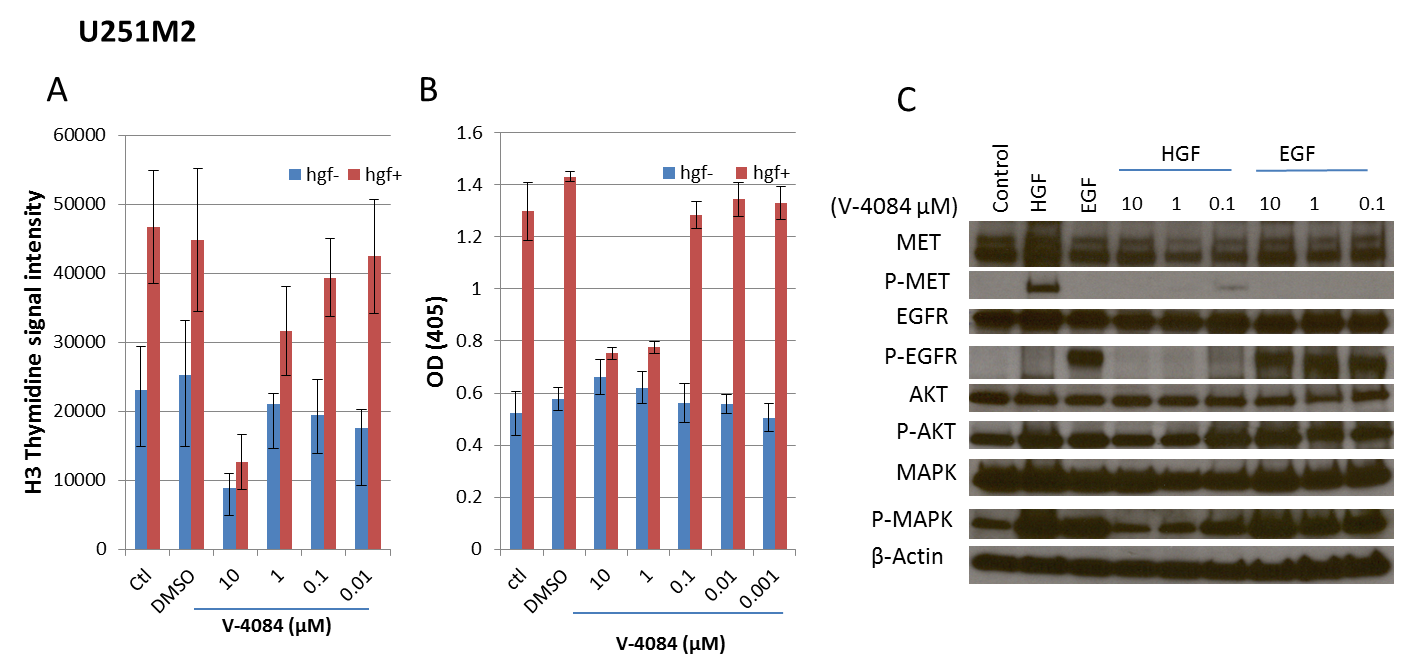
**

**Supplementary Figure 1. V-4084 inhibited HGF-paracrine-activated proliferation and invasion.**

1. [^3^H]-thymidine incorporation was used to test HGF-dependent proliferation *in vitro*. HGF at 100 ng/ml significantly stimulated DNA synthesis in U251M2 cells; such synthesis was dose-dependently inhibited by V-4084. Triplicates were used for each concentration. Short bar refers to standard deviation. * Comparing with control group, inhibition was found statistically significant (student *t* test, p<0.05).
2. Urokinase assay was used to test HGF-dependent invasion *in vitro*. HGF at 100 ng/ml significantly increased U251M2 invasion, which was inhibited by V-4084 dose-dependently. Triplicates were used for each concentration. Short bar refers to standard deviation.

* Comparing with control group, inhibition was found statistically significant (student *t* test, p<0.05).

1. V-4084 inhibited HGF-dependent (but not EGF-dependent) downstream AKT and MAPK pathways in U251M2 cells.


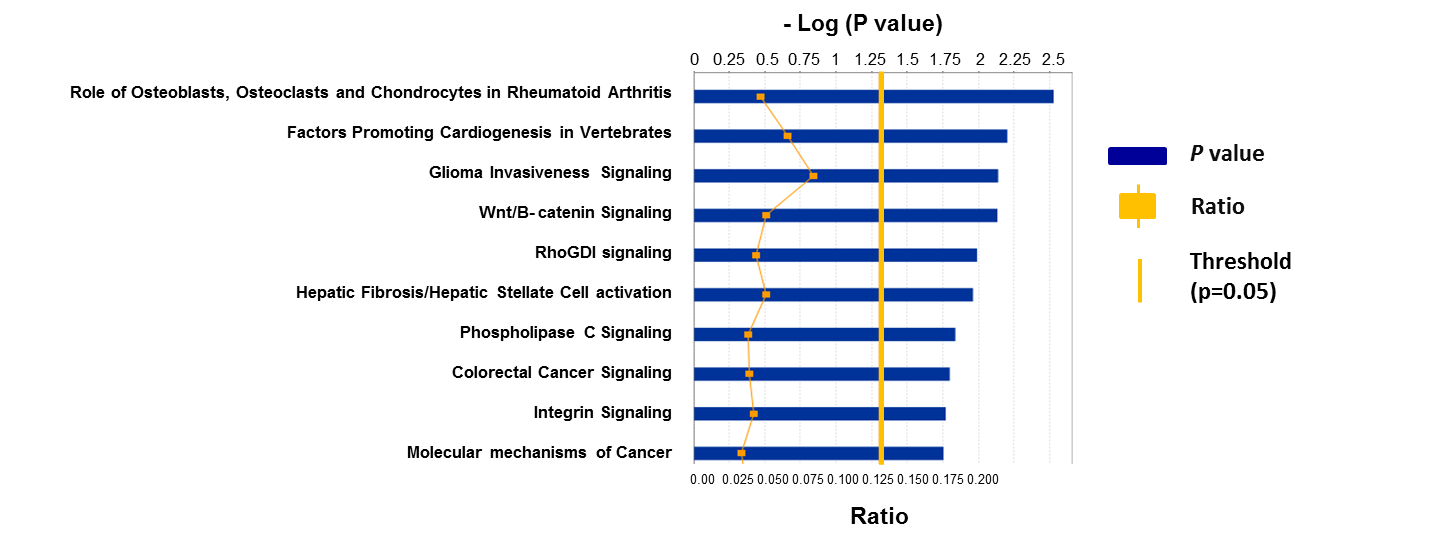


**Supplementary Figure 2. Top ten affected signaling pathways activated in HGF-autocrine tumors that are sensitive to MET inhibitors.**

Gene expression profiles of tumors sensitive and insensitive to V-4084 were analyzed and we identified 301 differentially expressed genes between the two phenotypes (Fig. 2C). Ingenuity Pathway Analysis (IPA) was used to determine the 10 most affected pathways. Note that the “Glioma Invasiveness Signaling” is third on the graph. The p value for each pathway is expressed as –log (P value); “ratio” = the number of the genes in a given pathway that meet the threshold ÷ total number of genes in the given pathway; “threshold” refers to the cutoff at p = 0.05.


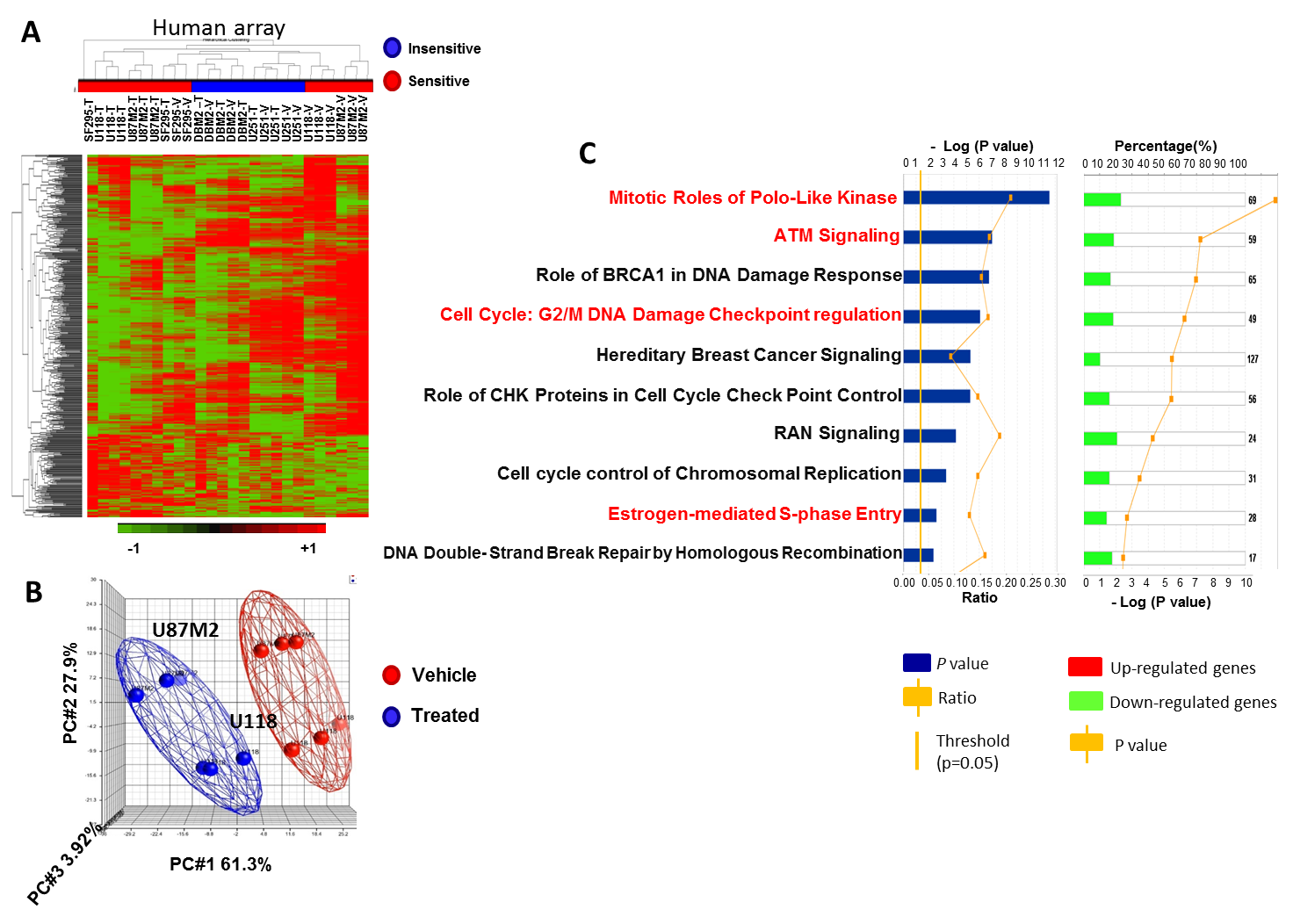


**Supplementary Figure 3. Gene expression changes in glioma xenograft tumors in response to V-4084 treatment determined by human array.**

(A) Supervised clustering of 485 genes derived from treated vs. vehicle samples from all GBM tumors, performed on the human array (Student’s *t*-test, p < 0.01).

(B) PCA analysis conducted on the 550 genes from treated vs. vehicle samples from only U87M and U118 tumors, performed on the human array (Student’s *t*-test, p < 0.01).

(C) IPA analysis of the 550 differentiated genes derived from treated vs. vehicle samples from only U87M2 and U118 tumors, performed on the human array (Student’s *t*-test, p < 0.01). The 10 most significant pathways are shown. The p value for each pathway is expressed as –log (P value); “ratio” and “threshold” are defined as in Supplementary Fig. 2. Pathways indicated in red are the common to those found in mouse array (Supplementary Fig.4).

**
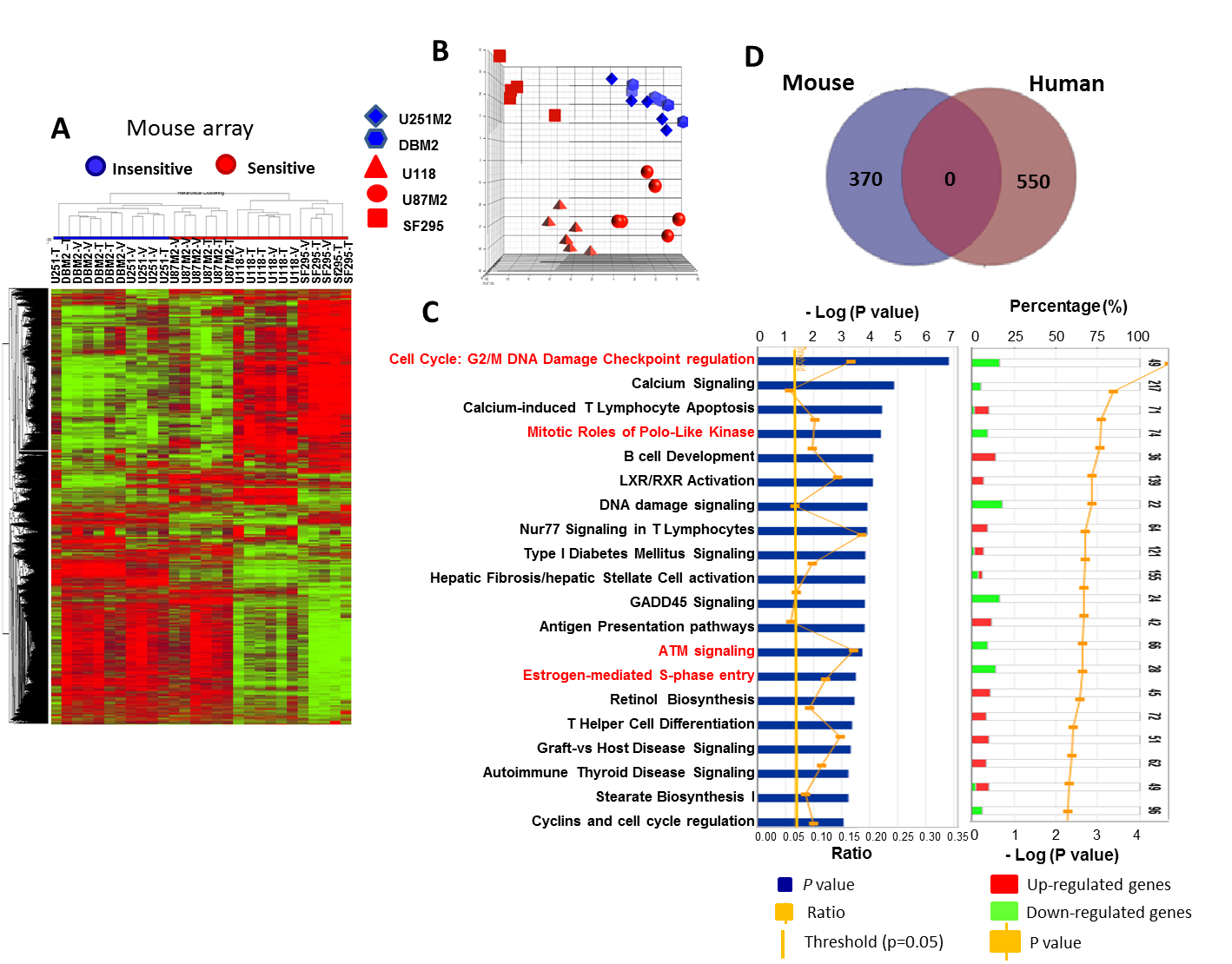
**

**Supplementary Figure 4. Gene expression changes in host cells within glioma cell line xenografts in response to V-4084 treatment determined by mouse array.**

A) Unsupervised cluster analysis based on the whole mouse gene data set showing good separation between sensitive and insensitive tumors.

B) PCA analysis using whole mouse gene expression data set showing good separation between sensitive and insensitive tumors.

C) IPA analysis using the same 370 genes derived from treated vs. vehicle samples of only U87M2 and U118 tumors (Student’s *t*-test, p < 0.01). The 20 most significant pathways are shown. The p value for each pathway is expressed as –log (P value); “ratio” and “threshold” are defined as in Suppl. Fig. 2. Pathways indicated in red are the common to those found in human array (Suppl. Fig.3)

D) Venn diagram showing no overlapping genes that were differentially expressed from the mouse (*n* = 370) or human arrays (*n* = 550).

**Supplementary Reference**

1. Gao C.F., Xie Q., Su Y.L. et al. Proliferation and invasion: plasticity in tumor cells. Proc Natl Acad Sci U S A*.* 2005;102(30):10528-33.

2. Webb C.P., Hose C.D., Koochekpour S. et al. The geldanamycins are potent inhibitors of the hepatocyte growth factor/scatter factor-met-urokinase plasminogen activator-plasmin proteolytic network. Cancer Res*.* 2000;60(2):342-9.

3. Xie Q., Bradley R., Kang L. et al. Hepatocyte growth factor (HGF) autocrine activation predicts sensitivity to MET inhibition in glioblastoma. Proc Natl Acad Sci U S A*.* 2012;109(2):570-5.
